# Improving the Accuracy of TOF LiDAR Based on Balanced Detection Method

**DOI:** 10.3390/s23084020

**Published:** 2023-04-15

**Authors:** Jingjing Li, Ying Bi, Kun Li, Lingyi Wu, Jie Cao, Qun Hao

**Affiliations:** 1 School of Optics and Photonics, Beijing Institute of Technology, Beijing 100081, China; 2 Beijing Institute of Aerospace Systems Engineering, Beijing 100076, China; 3 School of Mechatronical Engineering, Beijing Institute of Technology, Beijing 100081, China; 4 Yangtze Delta Region Academy, Beijing Institute of Technology, Jiaxing 314003, China; 5 School of Opto-Electronic Engineering, Changchun University of Science and Technology, Changchun 130013, China

**Keywords:** balanced detection, time-of-flight (TOF), accuracy, lidar

## Abstract

The ranging accuracy of pulsed time-of-flight (TOF) lidar is affected by walk error and jitter error. To solve the issue, the balanced detection method (BDM) based on fiber delay optic lines (FDOL) is proposed. The experiments are carried out to prove the performance improvement of BDM over the conventional single photodiode method (SPM). The experimental results show that BDM can suppress common mode noise and simultaneously shift the signal to high frequency, which reduces the jitter error by approximately 52.4% and maintains the walk error at less than 300 ps with a non-distorted waveform. The BDM can be further applied to silicon photomultipliers.

## 1. Introduction

Lidar based on pulsed time-of-flight (TOF) is widely used in industrial precision measurement [1], unmanned aerial vehicle mapping [2,3], military [4,5] and autonomous driving [6,7]. The key metric for pulsed TOF laser ranging is the accuracy of distance measurement [5]. However, the accuracy of the pulsed TOF laser ranging will be reduced due to ranging error, which is caused by various noise sources, detection circuit hardware, echo pulse rise time and other factors.

The ranging error mainly includes walk error and jitter error [8]. Walk error is primarily manifested by the timing point drift caused by the change in the amplitude of the echo pulse [9,10]. The constant ratio timing discrimination method can reduce a certain degree of the walk error caused by the amplitude change of the echo signal [11], but it will bring more circuit noise. It is possible to improve the ranging accuracy by using an analog-to-digital converter with a high sampling rate to sample the full waveform of the echo pulse signal [12], which will lead to the problem of power consumption. The method of compensating for the walk error by measuring the rise time or pulse width of the echo pulse signal can also be used to decrease the effect on the ranging accuracy. However, it requires to be calibrated and calculated separately, which increases the complexity of the lidar signal processing system and reduces its processing speed [13,14,15]. Another approach to reduce the walk error is to complete the shaping of the unipolar to bipolar pulses at the receiver input through an LC resonator, where the first zero-crossing point is used as the timing marker [16].

Jitter error is generally limited by the bandwidth and signal-to-noise ratio (SNR) of the receiver [9,17,18], which is a statistical error in the signal range due to noise and circuit nonlinearity [19,20,21]. There are many different types of noise, including background noise, shot noise brought on by laser signal, dark current noise, thermal noise, amplifier noise, etc. The background noise takes a great effect when measured, particularly in a bright environment [22,23]. When single-photon detection techniques are used, the jitter error can be reduced by using materials with low latency such as superconducting nanowire single-photon detectors (SNSPDs) [24]. Liang et al. [25] developed a high-performance GHz-gated InGaAs/InP APD that operated in the quasi-free-running mode, and adjusted the repetition frequency of the gate to reduce jitter error from 156 ps to 114 ps. When linear optical detection techniques are used, jitter error can be reduced by improving the SNR of the echo signal. With the differential optical path method for dual optical path detection, a part of the common mode noise generated by the light intensity can be eliminated to improve the SNR of the lidar system [26]. However, it makes the lidar system bulky and complex to assemble and calibrate, which is difficult to be applied in practical engineering.

From the above, we found that the ranging error is still the main issue of improving the ranging accuracy. Therefore, we propose a balanced detection method (BDM) based on fiber optic delay lines to restrain the noise in the detection. It can transform the unipolar pulse signal into the bipolar signal by a balanced photodetector (BPD) and fiber delay optic lines (FDOL), and take the zero-crossing point as the moment identification point to improve the ranging accuracy of lidar systems. In addition, the lidar system based on BDM will not introduce excessive power consumption, with its small size and compact structure, which is convenient in practical engineering.

This paper presents the working principle of the pulsed TOF lidar system based on BDM and the noise sources thereof. Then, the BDM and the traditional single photodetector method (SPM) are used to experiment on the response of background noise and laser signal, which show that the BDM can effectively suppress the common mode noise and make the laser signal energy more concentrated. Finally, we measured the jitter error and the walk error across the two methods under the same conditions. The results show that the BDM can improve the SNR, reducing the jitter error and the walk error, which effectively improves the ranging accuracy.

## 2. Methods

**Figure 1:**
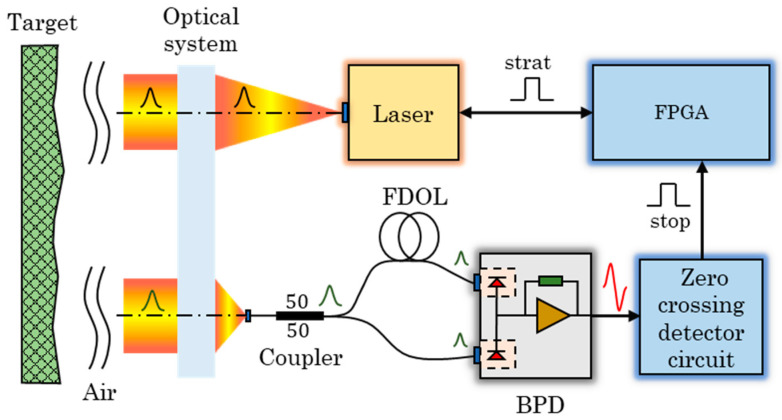
The block diagram of the pulsed TOF lidar system based on BDM.

The block diagram of the pulsed TOF lidar system based on BDM is shown in Figure 1. The working principle is as follows. The field programmable gate array (FPGA) triggers the laser to emit a short pulse laser, and marks this moment as the start moment of the TOF. The laser pulse is collimated by the optical system and then illuminated to the target. The echo pulse reflected by the target is converged by the optical system and coupled into a multimode fiber coupler with a 50:50 splitting ratio. One path is coupled into one side of the BPD via FDOL whose distance is the equivalent optical pulse width [26], and the other path is coupled directly into the other side of the BPD. The zero-crossing comparator identifies the moment of the differential analog signal from the BPD output and exports the digital signal representing the stop moment to the FPGA. The time of flight is calculated by the FPGA through the start moment and the stop moment.

The laser pulse is with a temporal function of the Gaussian model [27] and can be written as:

(1) $$P_{t}\left(t\right)=\frac{E_{t}}{\tau \sqrt{2\pi }}exp\left(\frac{-t^{2}}{2\tau^{2}}\right),$$

where $E_{t}$ is the original pulse energy and τ is the transmit pulse width. According to the lidar equation, the optical power $P_{r1}$ and $P_{r2}$ at the two sides of the BPD can be written as:

(2) $$\left\{\begin{array}{c} P_{r1}\left(t\right)=\frac{E_{t}T_{a}^{2}\eta_{t}\eta_{r}\rho_{r}D}{2\sqrt{2\pi }\tau }\cdot \exp\left[-\frac{1}{2\tau^{2}}{\left(t-\frac{2R+l}{c}\right)}^{2}\right]+P_{back1}\\ P_{r2}\left(t\right)=\frac{E_{t}T_{a}^{2}\eta_{t}\eta_{r}\rho_{r}D}{2\sqrt{2\pi }\tau }\cdot \exp\left[-\frac{1}{2\tau^{2}}{\left(t-\frac{2R}{c}\right)}^{2}\right]+P_{back2}\\ l=\frac{2c\tau }{n} \end{array}\right.,$$

where $T_{a}$ is the atmospheric transmittance, $\eta_{t}$ is the transmitting optical system efficiency, $\eta_{r}$ is the receiving optical system efficiency, $\rho_{r}$ is the target reflectivity, D is the optical lens diameter, R is the detection distance, l is the length of the fiber, n is the medium refractive index and $P_{back1}$ and $P_{back2}$ are the power of the background noise.

**Figure 2:**
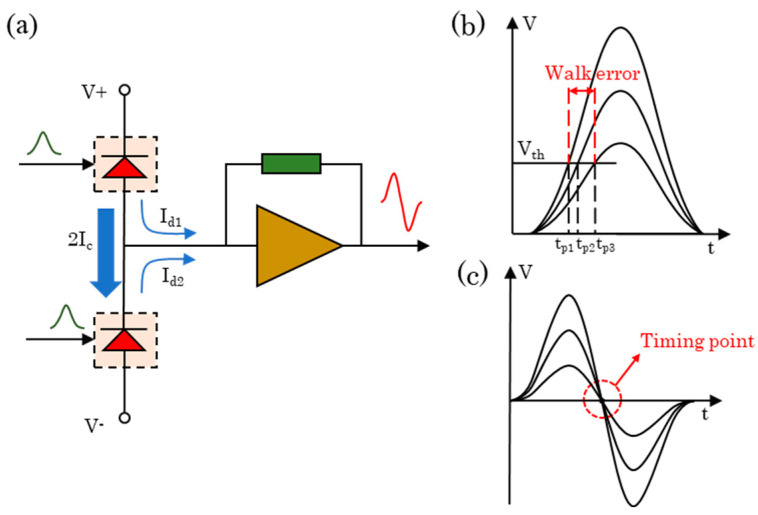
(**a**) The flow of photocurrent in the BPD. (**b**) Conceptual diagram of the walk error. (**c**) The bipolar signal with zero-crossing point as the timing moment.

The BPD mentioned above converts the input optical signal to photocurrent using two selected and well-matched photodiodes, with PD1 generating a photocurrent of $I_{c}+I_{d1}$ and PD2 generating a photocurrent of $I_{c}-I_{d2}$. As shown in Figure 2a, the current flow is divided into two parts: the same components of photocurrent $I_{c}$ in two channels form a loop to the ground and flow from V+ to V−, which suppresses the common mode noise.

The different components of photocurrent $I_{d1}$ and $I_{d2}$ in two channels flow into the transimpedance amplifier, which then becomes the output bipolar voltage signal. Walk error caused by variation in echo pulse amplitude during ranging can be as large as several nanoseconds [16], as shown in Figure 2b. The BDM takes the zero-crossing point of the bipolar signal as the timing moment, shown in Figure 2c, which can keep the walk error at a low level.

The jitter error of pulsed TOF laser ranging is roughly proportional to the rise time and the SNR of the echo pulse signal [17,28].

(3) $$\sigma_{R}\approx \frac{t_{r}}{SNR},$$

where $\sigma_{R}$ is the jitter error of the ranging, $t_{r}$ is the rise time of the pulse. In the condition of constant bandwidth, the improvement of SNR will reduce the jitter error. The expression of SNR can be written as:

(4) $$SNR=\frac{i_{S}}{i_{N}}=\frac{i_{S}}{\sqrt{\bar{i_{n,back}^{2}}+\bar{i_{n,dark}^{2}}+\bar{i_{n,shot}^{2}}+\bar{i_{n,th}^{2}}+\bar{i_{n,amp}^{2}}}},$$

where $i_{S}$ is the signal current and the denominator of the equation corresponds to various noise sources, $\bar{i_{n,back}^{2}}$ is the background noise, $\bar{i_{n,dark}^{2}}$ is the dark current noise of the photodetector, $\bar{i_{n,shot}^{2}}$ is the shot noise caused by the laser signal, $\bar{i_{n,th}^{2}}$ is the thermal noise and $\bar{i_{n,amp}^{2}}$ is the noise introduced by the amplifier circuit [29,30,31,32].

Compared with the conventional SPM, $\bar{i_{n,th}^{2}}$ and $\bar{i_{n,amp}^{2}}$ in both noise sources can be considered approximately equal when ensuring the same structure of the amplifier circuit behind the receiver circuit of BDM and SPM. A portion of the other three noise components is random numbers whose probability distribution functions obey the Poisson distribution [33,34]. Because of the strong consistency between the two diodes for manufacturing the BPD [35], the common mode noise can be effectively suppressed during the differential processing of the photocurrent. It provides a certain degree of suppression to these three types of noise and especially background noise, thus improving the SNR.

## 3. Experiment and Results

In order to test the performances of BDM in improving the accuracy of pulsed TOF laser ranging, and also to compare the differences between the BDM and the traditional SPM, we carried out experiments in three aspects as follows: Firstly, the response of the two methods to the background noise and the suppression effect of BDM on the background noise was tested. Meanwhile, we studied the influence of differential extension distance on the effectiveness of BDM to suppress background noise. Then, we compared the laser echo pulses obtained with SPM and BDM to study the characteristics of the laser signal in the power spectrum. Finally, the jitter error and the walk error of the ranging based on SPM and BDM were tested.

### 3.1. Suppressing the Effect of Background Noise

**Figure 3:**
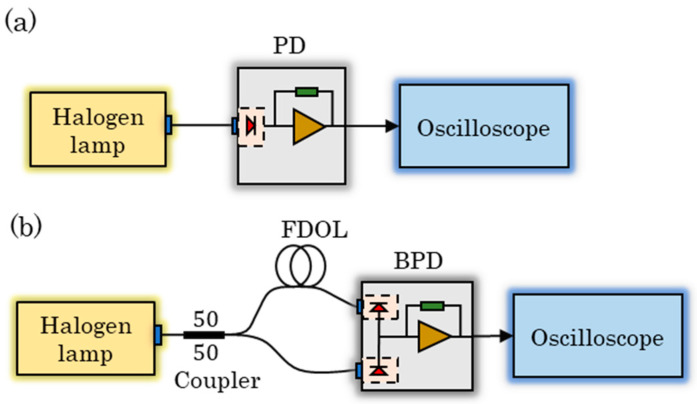
The experimental setup to measure the background noise: (**a**) based on SPM and (**b**) based on BDM.

In order to observe the suppression effect of BDM on background noise, the experimental platform shown in Figure 3 was constructed.

To begin with, we measured the self-noise of the circuit using separately two methods without the background noise introduced by the environmental light and found it to be almost identical. Then, as shown in Figure 3a, we used a halogen lamp (MR16 13,163 24 V 250 W GX5.3) with a color temperature of 3300 K–5000 K to provide the background noise, and the background light is directly coupled into the PD through the multimode fiber optic patch cable (XFOPC-1064mm-0-FA) thus obtaining the background noise measured by SPM. According to the experimental setup shown in Figure 3b, the same background noise is coupled into a multimode 1 × 2 fiber coupler with a splitting ratio of 50:50 (YXOS-1064sm-1*2-0-FA). One way is coupled into the BPD through the differential extension line (length of 1 m) composed of multimode fiber optic patch cables, and the other way is directly coupled into the BPD. The background noise measured by BDM is thus obtained.

**Figure 4:**
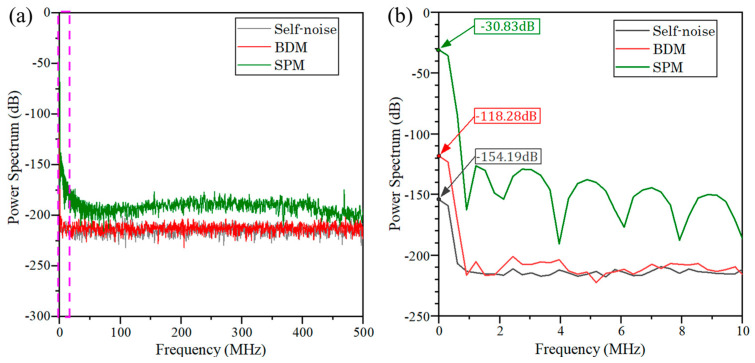
Power spectra of background noise and circuit self-noise measured by the two methods over the frequency band of (**a**) 0~500 MHz and (**b**) 0~10 MHz.

The results are shown in Figure 4, showing the power spectra of the circuit self-noise (gray), the background noise measured by SPM (green), and BDM (red), which shows that the BDM can effectively suppress the background noise. It can be seen that the BDM can reduce the direct current (DC) component of the background noise by about 87 dB compared to the traditional SPM with the background light noise applied. Meanwhile, the BDM can suppress the alternate current (AC) noise caused by the background light by about 30 dB~87 dB in the range of 0~10 MHz.

**Figure 5:**
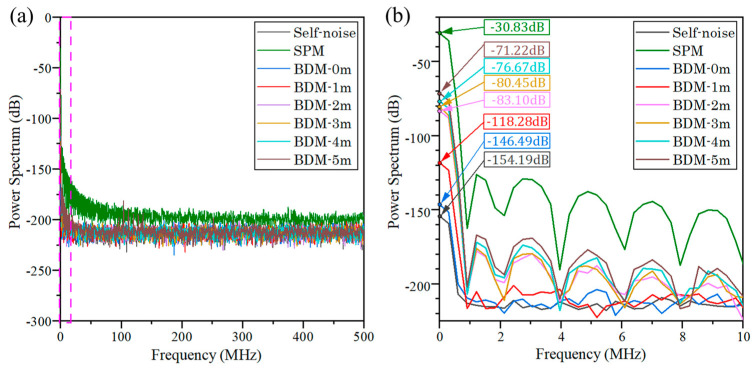
Power spectra of circuit self-noise and background noise measured by BDM with different lengths of differential extension lines in the frequency band of (**a**) 0~500 MHz and (**b**) 0~10 MHz.

In addition to measuring the circuit self-noise and the response of the two methods to background noise, we also explored the influence of the FDOL on the background noise suppression effect of BDM. We performed the power spectrum analysis of the background noise measured by BDM at 0 m, 1 m, 2 m, 3 m, 4 m and 5 m with the same background noise applied, where the length of the differential extension was changed by changing the number of multimode fiber optic patch cables. The results are shown in Figure 5, which can see that the BDM can reduce the DC component of the background noise by about 40~87 dB compared to the conventional SPM when the differential extension line length is 1 m~5 m. It can also perform a certain effect of suppressing the AC noise in the range of 0~10 MHz caused by the background light. When the differential extension line length is 0 m, the common mode component of the background noise is stronger, and the suppression effect of the DC component of the noise is the best, which can reach 115 dB. With the increase in the differential extension line length, the suppression effect of BDM on the background noise gradually decreases and tends to 40 dB.

### 3.2. Responsivity

**Figure 6:**
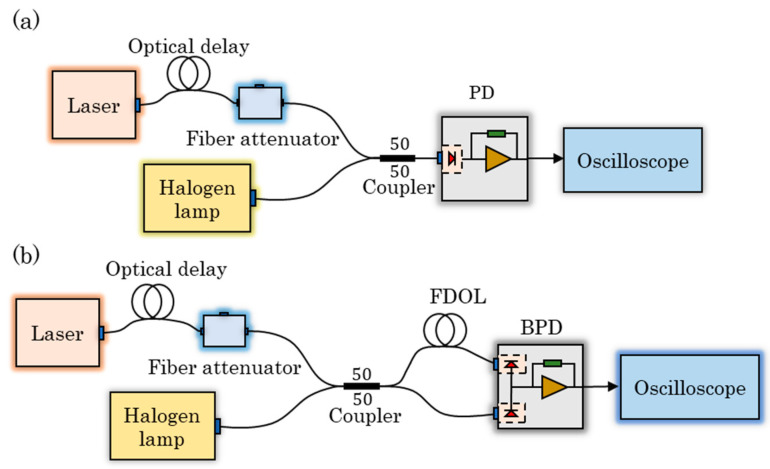
The experimental setup to measure the laser signal: (**a**) based on BDM and (**b**) based on SPM.

In order to observe the difference between the laser echo signal measured by BDM and traditional SPM, we constructed an experimental setup as shown in Figure 6.

The laser (VLSS-1064-M-PL, Connet, Shanghai, China) is set in internal trigger mode with a pulse width of 3 ns, a re-frequency of 1000 kHz, and a peak output power of 100 mW. As shown in Figure 6a, the laser emitted from the laser is coupled into the fiber coupler with a 50:50 splitting ratio after passing through the fiber extension and the electrically adjustable fiber attenuator (YXVOA-1064sm-MVOA-0-FA, Yixun Photon, Mianyang, China). At the same time, the background noise provided by the halogen lamp is also coupled to the fiber coupler. Then, the mixed laser signal and background noise are coupled into the PD simultaneously so that the response of SPM to the laser pulse signal is obtained. According to the experimental setup shown in Figure 6b, we couple the same laser signal and background noise into the multimode 2 × 2 fiber coupler (TM200R5F2B, Thorlabs, Newton, NJ, USA) with a 50:50 splitting ratio. Then, the mixed laser signal and the background noise are divided into two ways and coupled into the two detectors of the BPD to obtain the response of the BPD method to the laser pulse signal.

**Figure 7:**
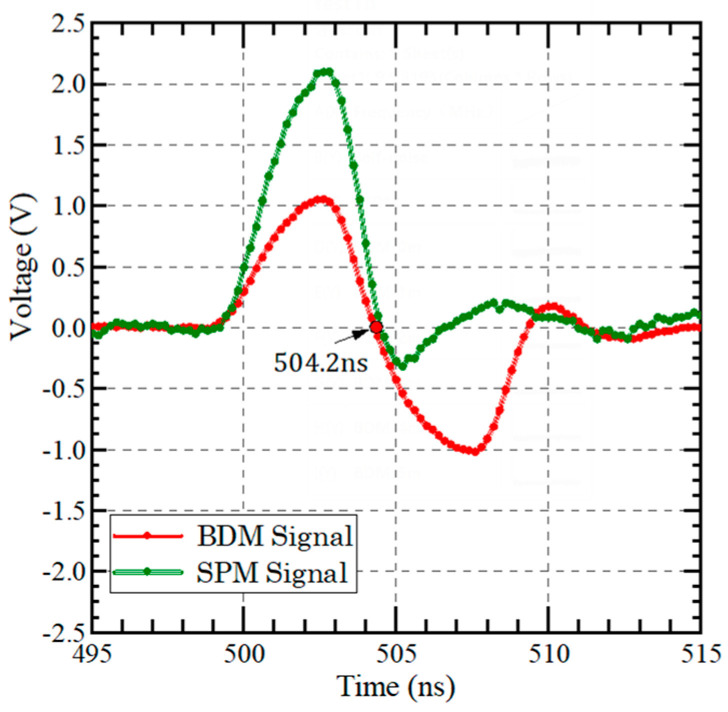
Comparison of the signals acquired by SPM and BDM.

The waveforms of the laser signals measured by SPM and BDM are shown in Figure 7. It can be seen that the BDM converts the unipolar pulse signal into a bipolar pulse signal, and its crossing zero point is the stop moment of the TOF.

**Figure 8:**
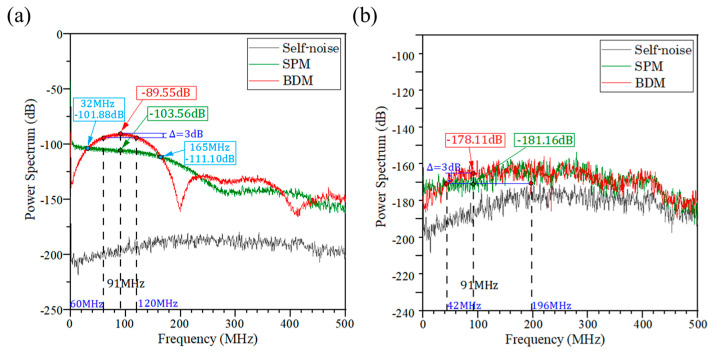
Power spectra of the signals sampled by the two methods for laser output peak powers of (**a**) 100 mW and (**b**) 5 mW.

Under the condition of keeping the same background light intensity, and setting the laser output peak laser power to 5 mW and 100 mW, we used SPM and BDM for detection based on the above experimental platform, and analyzed the power spectrum of the obtained signal, which is shown in Figure 8.The signal measured by SPM is a typical Gaussian signal with the highest intensity at a low frequency in the spectrum and decreasing as the frequency increases, while the signal of BDM is relatively concentrated around 91 MHz. It can be seen that the laser signal measured by BDM shifted to a high frequency in the spectrum, and the signal intensity of BDM at 91 MHz is 3.05 dB and 14.01 dB stronger than that of SPM at the same frequency, respectively. While the background noise is mainly concentrated in the low frequency, the signal moves to the high frequency, which is more favorable to distinguish the effective signal.

As can be seen from the above experiments, compared to the SPM, the BDM provides excellent suppression of noise, especially background light noise, as well as concentrating the energy of the laser signal to a higher frequency.

### 3.3. Improvement on Jitter Error and Walk Error

**Figure 9:**
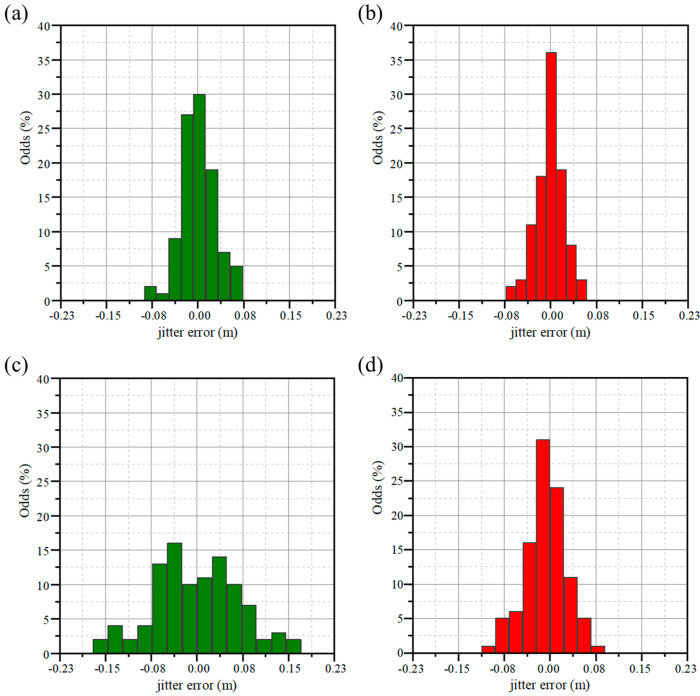
Jitter error under high SNR conditions based on (**a**) SPM and based on (**b**) BDM and under low SNR conditions based on (**c**) SPM and based on (**d**) BDM.

Based on the experimental setup shown in Figure 6, we set the halogen lamp to provide the same background noise and measured the jitter error of both methods in high SNR conditions and low SNR conditions, respectively. We adjusted the voltage of the fiber attenuator so that the signal SNR detected by SPM was approximately equal to 9. Under this condition, we used BDM and SPM to range 1000 times continuously, and obtained the ranging error of the two methods. Then we adjusted the SNR to be approximately equal to 2 and repeated the above experiments to measure the ranging error of the two methods in low-SNR conditions, as shown in Figure 9.

When the ranging experiment was carried out in high-SNR conditions, the root-mean-square (RMS) value of the jitter error for SPM ranging was calculated to be 184 ps, while the jitter error for BDM ranging was 154 ps. In this case, the jitter error of BDM was reduced by 16% compared to SPM. According to Equation (3), the effect of jitter error is more significant when ranging in a low SNR condition. When ranging in a low SNR condition, the RMS value of the jitter error for SPM ranging was calculated to be 462 ps, while the jitter error for BDM ranging was 220 ps. In the case of low SNR, the BDM can reduce the jitter error by 52.4%.

**Figure 10:**
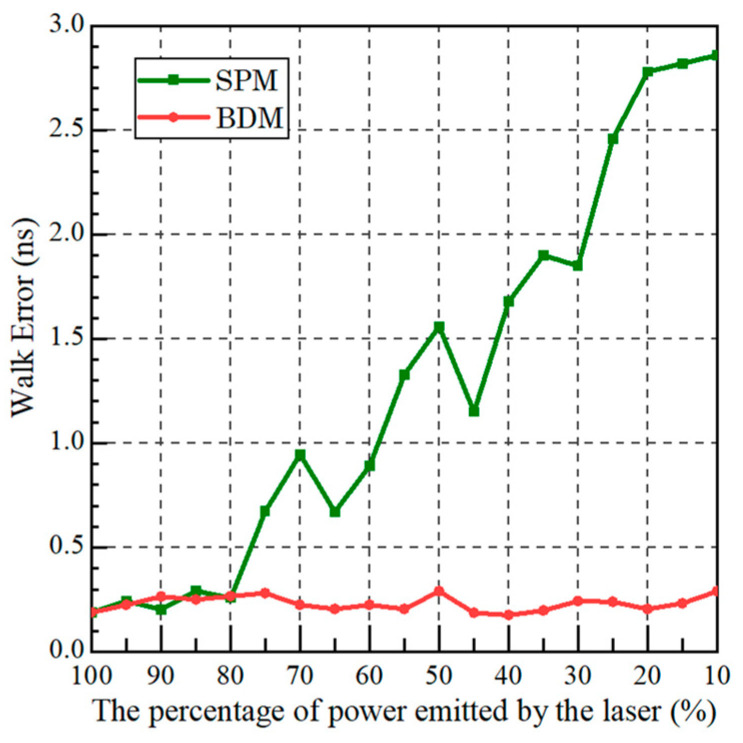
RMS of walk error under different laser powers based on the two method measurements.

In order to observe the improvement effect of BDM on walk error, we conducted several ranging experiments using the experimental setup in Figure 6. The halogen lamp was used to provide the background noise and the peak output power of the laser was 200 mW. We changed the laser power by adjusting the fiber attenuator and recorded the RMS values of SPD and BDM ranging error without distortion of the waveform, as shown in Figure 10.

It can be seen that the ranging error of SPM gradually increases as the laser signal power decreases, while the ranging error of BDM does not change much and remains relatively stable between 200 ps~300 ps, indicating that the BDM can effectively suppress the walk error.

## 4. Conclusions

In summary, we propose a BDM based on fiber optic delay lines to the accuracy of the pulsed TOF laser range, and experimentally verify its effectiveness in improving SNR and reducing the walk error and the jitter error. The experimental results illustrate that the BDM can effectively suppress the common mode part of the noise such as background noise compared with the traditional SPM. The laser signal can be shifted to a higher frequency in the power spectrum to make the signal energy more concentrated, which is beneficial to extract the effective signal and finally improve the SNR. Moreover, the BDM can reduce the jitter error to 52.4% of the SPM, and has a significant reduction effect on the walking error. Meanwhile, the BDM is small in size and simple in structure, which has a good application prospect in engineering practice and can be further promoted for applications such as single photon measurement.

## Data Availability

Data underlying the results presented in this paper are not publicly available at this time but may be obtained from the authors upon reasonable request.
